# Leucine imparts cardioprotective effects by enhancing mTOR activity and mitochondrial fusion in a myocardial ischemia/reperfusion injury murine model

**DOI:** 10.1186/s13098-021-00755-z

**Published:** 2021-11-20

**Authors:** Atsushi Morio, Rie Tsutsumi, Shiho Satomi, Takashi Kondo, Hirotsugu Miyoshi, Takahiro Kato, Masashi Kuroda, Tadahiro Kitamura, Kenta Hara, Noboru Saeki, Hiroshi Sakaue, Yasuo M. Tsutsumi

**Affiliations:** 1 Department of Anesthesiology and Critical Care, Hiroshima University, 1-2-3 Kasumi, Minami, Hiroshima 734-8551 Japan; 2 Department of Nutrition and Metabolism, Institute of Biomedical Sciences, Tokushima University, 3-18-15 Kuramoto, Tokushima, 770-8503 Japan; 3 Laboratory of Metabolic Signal, Institute for Molecular and Cellular Regulation, Gunma University, 3-39-15 Showa-machi, Maebashi, Gunma 371-8512 Japan; 4 Kita Harima Medical Center, 926-250 Ichiba, Ono, Hyogo 675-1392 Japan

**Keywords:** Leucine, High-fat diet, Myocardial ischemia/reperfusion injury, Cardioprotective

## Abstract

**Background:**

Coronary artery disease is a leading cause of morbidity and mortality among patients with diabetes. Previously, we demonstrated that branched-chain amino acids (BCAAs) showed cardioprotective effects against cardiac ischemia/reperfusion (I/R) injury. A recent study suggested that leucine (Leu), a BCAA, is a key amino acid involved in mammalian target of rapamycin (mTOR) activity and mitochondrial function. However, whether Leu has cardioprotective effects on diabetic hearts is unclear. In this study, we examined the preconditioning effect of Leu treatment on high-fat diet (HFD)-induced obese mouse which simulate prediabetic heart.

**Methods:**

In vivo mice models of I/R injury were divided into the following groups: control, mTOR^+/−^, and high-fat diet (HFD)-induced obese groups. Mice were randomly administered with Leu, the mTOR inhibitor rapamycin (Rap), or Leu with Rap. Isolated rat cardiomyocytes were subjected to simulated I/R injury. Biochemical and mitochondrial functional assays were performed to evaluate the changes in mTOR activity and mitochondrial dynamics caused by Leu treatment.

**Results:**

Leu-treated mice showed a significant reduction in infarct size when compared with the control group (34.8% ± 3.8% vs. 43.1% ± 2.4%, n = 7, p < 0.05), whereas Rap-treated mice did not show the protective effects of Leu. This preconditioning effect of Leu was attenuated in mTOR^+/−^ mice. Additionally, Leu increased the percentage of fused mitochondria and the mitochondrial volume, and decreased the number of mitochondria per cell in isolated cardiomyocytes. In HFD-induced obese mice, Leu treatment significantly reduced infarct size (41.0% ± 1.1% vs. 51.0% ± 1.4%, n = 7, p < 0.05), which was not induced by ischemic preconditioning, and this effect was inhibited by Rap. Furthermore, we observed enhanced mTOR protein expression and mitochondrial fusion with decreased reactive oxygen species production with Leu treatment in HFD-induced obese mice, but not in mTOR^+/−^ mice.

**Conclusions:**

Leu treatment improved the damage caused by myocardial I/R injury by promoting mTOR activity and mitochondrial fusion on prediabetic hearts in mice.

## Introduction

Coronary artery disease (CAD) is a leading cause of morbidity and mortality among patients with diabetes, which increases the risk of developing CAD by two- to fourfold [1, 2]. Both type 1 and type 2 diabetic individuals are prone to developing ischemic heart disease, including acute myocardial infarction (AMI) and post-infarct complications [3]. Mortality from AMI is approximately twice among diabetic patients compared with that among non-diabetic individuals [4, 5]. Despite the burden of ischemic heart disease among patients with diabetes, effective treatment is currently unavailable [6].

Myocardial ischemia/reperfusion (I/R) injury is a condition wherein the damage is caused by the occlusion of coronary arteries and restoration of blood flow to the ischemic myocardium [7, 8]. Several studies have reported the preconditioning effect of pharmacological agents through signaling pathways regulating cellular processes such as necrosis, apoptosis, and autophagy [9–11].

Recently, we showed the cardiac preconditioning effect of branched-chain amino acid (BCAA) treatment on I/R injury in mice, resulting from increased mammalian target of rapamycin (mTOR) activity and improved mitochondrial function [12]. Among BCAAs, leucine (Leu) can activate mTOR kinase, thereby leading to the phosphorylation of p70S6 kinase and increasing the phosphorylation of serine residues in insulin receptor substrate-1 [13], which inhibits insulin signaling and insulin-stimulated glucose transport in muscles and fats. However, whether Leu has cardioprotective effects on diabetic hearts is unclear.

In this study, we examined the changes in mTOR activity and mitochondrial dynamics caused by Leu administered during I/R injury of the heart in a high-fat diet (HFD)-induced obese mouse model.

## Materials and methods

### Animals

All animals were treated in compliance with the guidelines for proper conduct of animal experiments and related activities (Ministry of Education, Culture, Sports, Science, and Technology of Japan). Additionally, the protocols, which follow the ARRIVE guidelines [14], were approved by the Animal Care and Use Committee at the University of Tokushima. Adult male Wistar rats and male C57BL/6 mice at 4 weeks of age were purchased from Japan SLC, Inc. (Shizuoka, Japan). mTOR^+/−^ mice were created as previously reported [15]. Mice were housed under temperature- (23 ± 3 ℃) and humidity-controlled conditions with a 12 h light/12 h dark cycle. Mice had free access to water and a control diet (14% of calories from fat; Oriental Yeast Co., Ltd., Tokyo, Japan) or an HFD (60% of calories from fat; Oriental Yeast Co., Ltd.). Mice were randomly assigned to 6 weeks of HFD or control diets, and the experiments are performed on them at 10 weeks of age that is considered a juvenile. At the time of 10 weeks, body weight of mice was wild-type mice; 26.4 ± 0.6 g, HFD-induced obese mice; 32.1 ± 1.1 g, mTOR^+/−^ mice; 26.3 ± 0.8 g (mean ± SD).

### In vivo myocardial I/R experiments

The surgical methods used were similar to those described previously [16–18]. Mice were anesthetized with sodium pentobarbital (80 mg/kg, i.p.) and mechanically ventilated with 100% oxygen using a pressure-controlled ventilator (TOPO ventilator, Kent Scientific Co., Torrington, CT, USA). The core body temperature was maintained with a heating pad, and electrocardiogram leads were placed to record the heart rate. An intercostal thoracotomy was performed to expose the heart. Ischemia was induced by occluding the left coronary artery (LCA) with a 7–0 silk suture for 30 min, after which the ligature was released, and the heart was perfused for 2 h. Mice were randomly assigned to each experimental group. Saline (0.9%) or Leu (200 mg/kg, IV) was administered 20 min before the occlusion. In the ischemic preconditioning (IPC) group, IPC was induced by occluding the LCA for 5 min, followed by 15 min of reperfusion immediately before the I/R procedure. After reperfusion, mice were heparinized, and the LCA was again occluded. The area at risk (AAR) was determined by staining with 1% Evans blue (1.0 mL, Sigma). The heart was immediately excised and cut into 1.0-mm slices (McIlwain tissue chopper; Brinkmann Instruments). Each slice of left ventricle was then counterstained with 2,3,5, triphenyltetrazolium chloride (Sigma). After overnight storage in 10% formaldehyde, slices were weighed and visualized under a microscope (SZ61-TR, Olympus) equipped with a charge coupled device camera (DXM 1200F, Nikon). The images were analyzed (Image-Pro Plus, Media Cybernetics), and AAR and infarct size were determined by planimetry as previously described [8].

### Isolation and maintenance of rat cardiomyocytes

Cardiomyocytes were isolated from adult male Wistar rats. Rats were heparinized (1.0 IU/g, i.p.) 30 min before anesthetizing them with pentobarbital (80 mg/kg, i.p.). Myocytes were obtained via enzymatic (210 U/mg collagenase II; Worthington, Lakewood, NJ, USA) digestion of the heart using a Langendorff apparatus. Enzymatic digestion was performed as previously described [19, 20]. Isolated myocytes were then cultured in 4% fetal bovine serum on laminin (2 μg/cm^2^)-coated plates for 1 h. Culturing/maintenance media were changed to serum-free media [1% bovine serum albumin + 0.1% penicillin/streptomycin M199 media (Invitrogen, Carlsbad, CA, USA)] to eliminate all non-myocytes, and cardiac myocytes were incubated at 37 ℃ in 5% CO_2_ for 24 h.

### Mitochondrial dynamics analysis in isolated rat cardiomyocytes

Six hours prior to the pretreatment, all media were replaced with amino acid-free Dulbecco’s Modified Eagle’s medium to wash out any amino acids in M199 media. L-Leu (2.3 g; Sigma-Aldrich, St. Louis, MO, USA) was dissolved in 100 mL distilled water, creating a 175 mM stock solution. Cells were pretreated with Leu (160 µM) or control [phosphate-buffered saline (PBS)] for 2 h. We evaluated the changes in mitochondrial dynamics of each group for 4 h at 30 min intervals after the administration of Leu or PBS.

After pretreatment with Leu or PBS, simulated ischemia/reperfusion was performed. Simulated ischemia was induced by replacing the air content with a 95% N_2_ and 5% CO_2_ gas mixture at 2 L/min in a chamber and the media with glucose-free media. This was performed for 60 min, followed by 60 min of “reperfusion” by replacing the media with normal maintenance media and by incubating the cells with 21% O_2_ and 5% CO_2_. Finally, the cells were maintained in Krebs solution and fixed with 4% paraformaldehyde after incubating them for 30 min with MitoTracker Green FM (400 nmol/L; Molecular Probes, Invitrogen). Confocal image stacks were captured using a Leica laser microscope (Leica, Tokyo, Japan), as described previously [21]. Mitochondrial density was quantified using the ImageJ software (NIH, Bethesda, MD, USA). The number and volume of each mitochondrion were quantified using the Image J 3D Object Counter plug-in. The percentage of cells with a fusion pattern was determined based on the criteria that evaluated mitochondrial fusion with mitochondrial volume and a decrease in the number of mitochondria [22, 23].

### Reactive oxygen species (ROS)

To measure ROS production in the myocardium, the OxiSelect™ in vitro ROS/RNS Assay kit (Cell Biolabs, San Diego, CA, USA) was used. Before the I/R procedure, mice were injected with 200 μL of saline, Leu, or Leu and rapamycin (Rap) into the right atrium. Hearts were excised immediately after the I/R procedure, and the measurement of ROS were performed according to the manufacturer’s instructions. The ROS content was determined using the predetermined dichlorodihydrofluorescein standard curve, and mean fluorescence units were recorded.

### Western blotting

Lysates were separated using sodium dodecyl sulfate–polyacrylamide gel electrophoresis on 10% polyacrylamide precast gels (Invitrogen, Carlsbad, CA, USA) and transferred to polyvinylidene difluoride membranes through electroelution. Membranes were blocked in 20 mM Tris-buffered saline with 1% Tween containing 5% skimmed milk and incubated with primary antibodies overnight at 4 ℃. Immunolabeled blots were visualized using horseradish peroxidase-conjugated secondary antibodies (Santa Cruz Biotechnology, Santa Cruz, CA, USA) and enhanced chemiluminescence reagent (GE Healthcare, Waukesha, WI, USA) [24].

### Electron microscopy

Whole hearts or cardiomyocytes were fixed with 2.5% glutaraldehyde in 0.1 M cacodylate buffer for 2 h at room temperature, post-fixed with 1% osmium tetroxide in 0.1 M cacodylate buffer for 1 h at room temperature, and embedded as monolayers using LX‐112 embedding kits (Ladd Research, Williston, VT, USA). Sections were stained with uranyl acetate and lead citrate, and observed under an electron microscope (EM). Random sections were taken by an EM technician who was blinded to the treatments [25].

### Statistical analyses

All results were analyzed by observers who were blinded to the animal treatment history. Data are presented as mean ± standard deviation. Differences between the treatment groups were tested for statistical significance by one-way analysis of variance, followed by Bonferroni’s post hoc test. Differences were considered significant at p < 0.05.

## Results

### Leu reduces infarct size in wild-type but not in mTOR^+/−^ mice

**Fig. 1:**
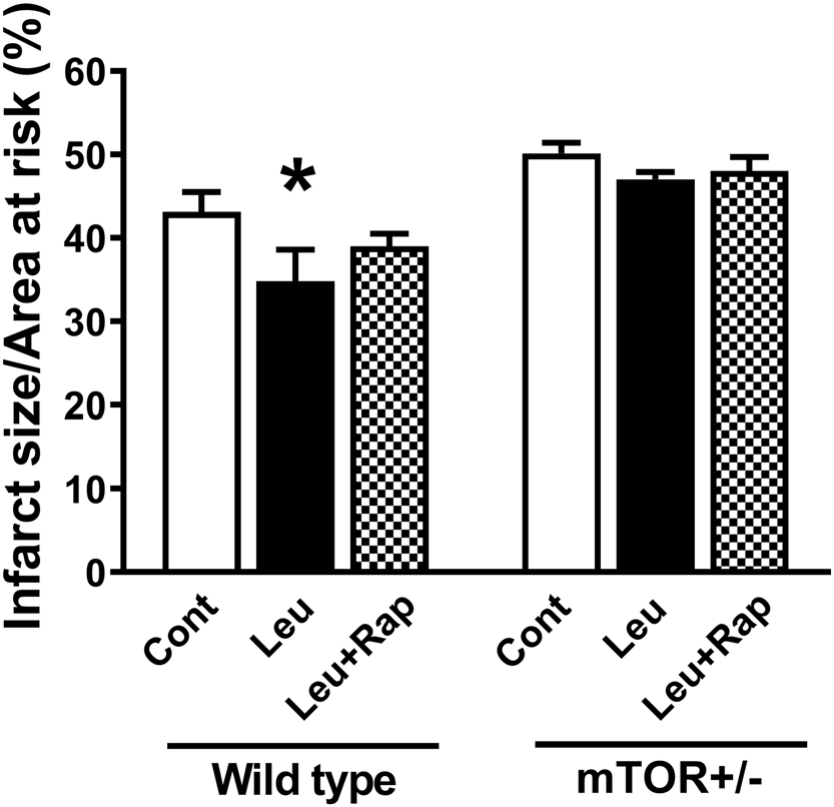
Effects of leucine (Leu) on myocardial ischemia/reperfusion (I/R) injury in mice. Leu reduced infarct size after I/R injury in wild-type mice. The protective effect of Leu was attenuated by rapamycin (Rap). Leu did not improve the infarct size in mTOR^+/−^ mice *p < 0.05, vs. control (Cont). mTOR, mammalian target of rapamycin

The area at risk was calculated as a percentage of infarct size, and was found to be similar between the groups. In mice treated with Leu, a significant reduction in myocardial I/R injury was observed when compared with wild-type mice treated with vehicle control (34.8% ± 3.8% vs. 43.1% ± 2.4%, n = 7, p < 0.05), but not in those treated with Leu and Rap. In addition, the effect of Leu was inhibited in mTOR^+/−^ mice (Fig. 1).

### Leu promotes mitochondrial fusion in isolated rat cardiomyocytes

**Fig. 2:**
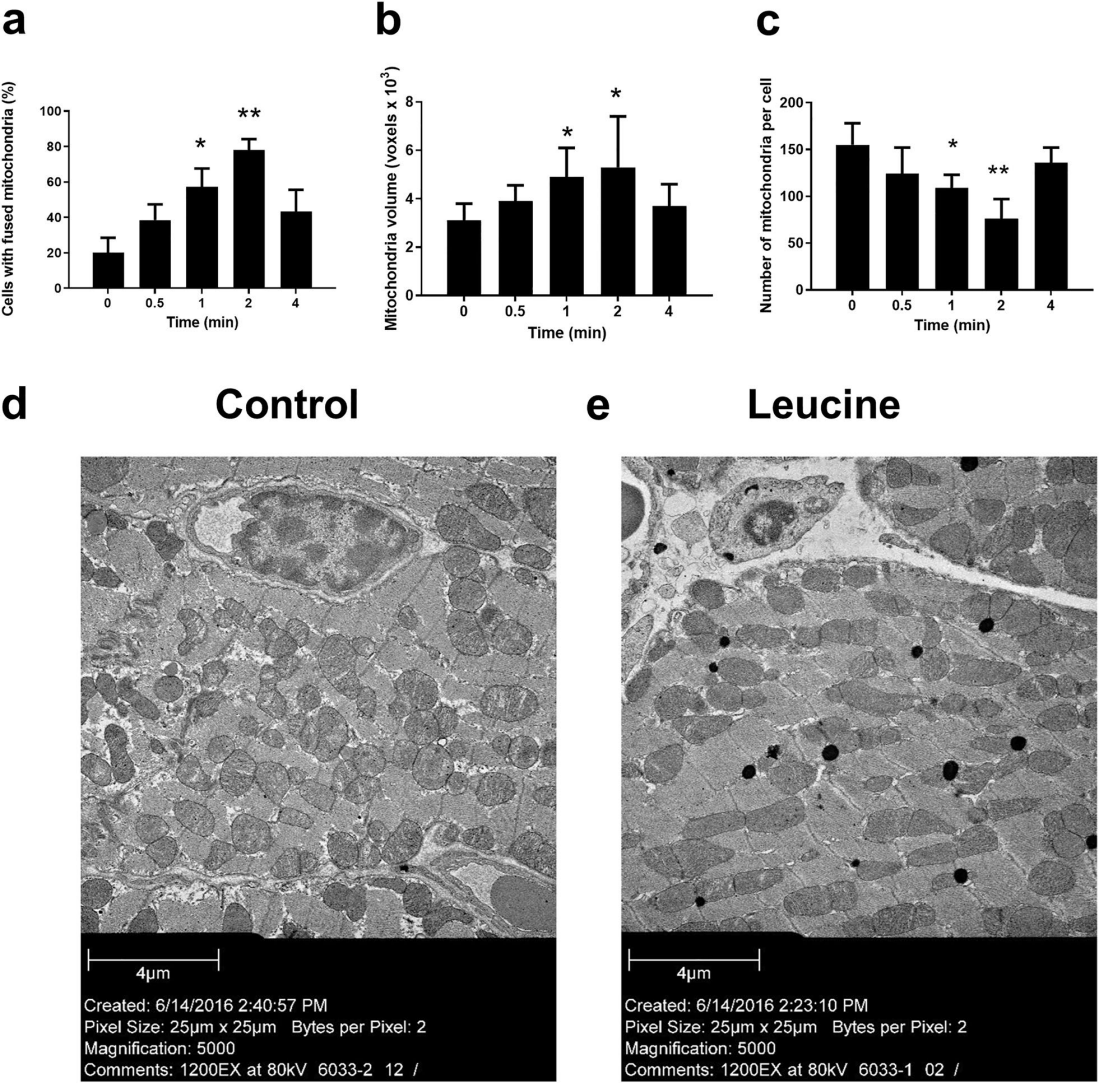
Effects of leucine (Leu) on mitochondria fusion in cardiomyocytes. The percentage of cells displaying **a** fused mitochondria and the **b** mitochondrial volume and **c** the number of mitochondria per cell were evaluated for 4 h at 30 min intervals after Leu pretreatment on isolated rat cardiomyocytes. The result of time 0 was used as the baseline. Leu led to an increase in mitochondrial fusion and mitochondrial volume and a decrease in the number of mitochondria after 2 h of incubation. *p < 0.05, **p < 0.01 vs. control (Cont). **d** Leu treatment (160 μM) for 2 h caused a decrease in the number of mitochondria and an increase in mitochondrial fusion in cardiomyocytes

In cardiomyocytes treated with Leu, mitochondria appeared to be interconnected. The percentage of cells displaying fused mitochondria was significantly increased at 2 h after Leu pretreatment when compared with the percentage of cells without Leu pretreatment (20% ± 8.5% vs. 78% ± 6.2%, n = 5, p < 0.01) (Fig. 2a). The volume of individual mitochondria was measured using the three-dimensional reconstitution of confocal stacks. Leu caused a 71% increase in the volume of individual mitochondria at 2 h after Leu treatment (Fig. 2b). In addition, Leu stimulated mitochondrial fusion with an increase in mitochondrial size, whereas the number of mitochondria per cell was found to be decreased (Fig. 2c, d).

### Leu reduces infarct size in HFD-induced obese mice through mTOR activity

**Fig. 3:**
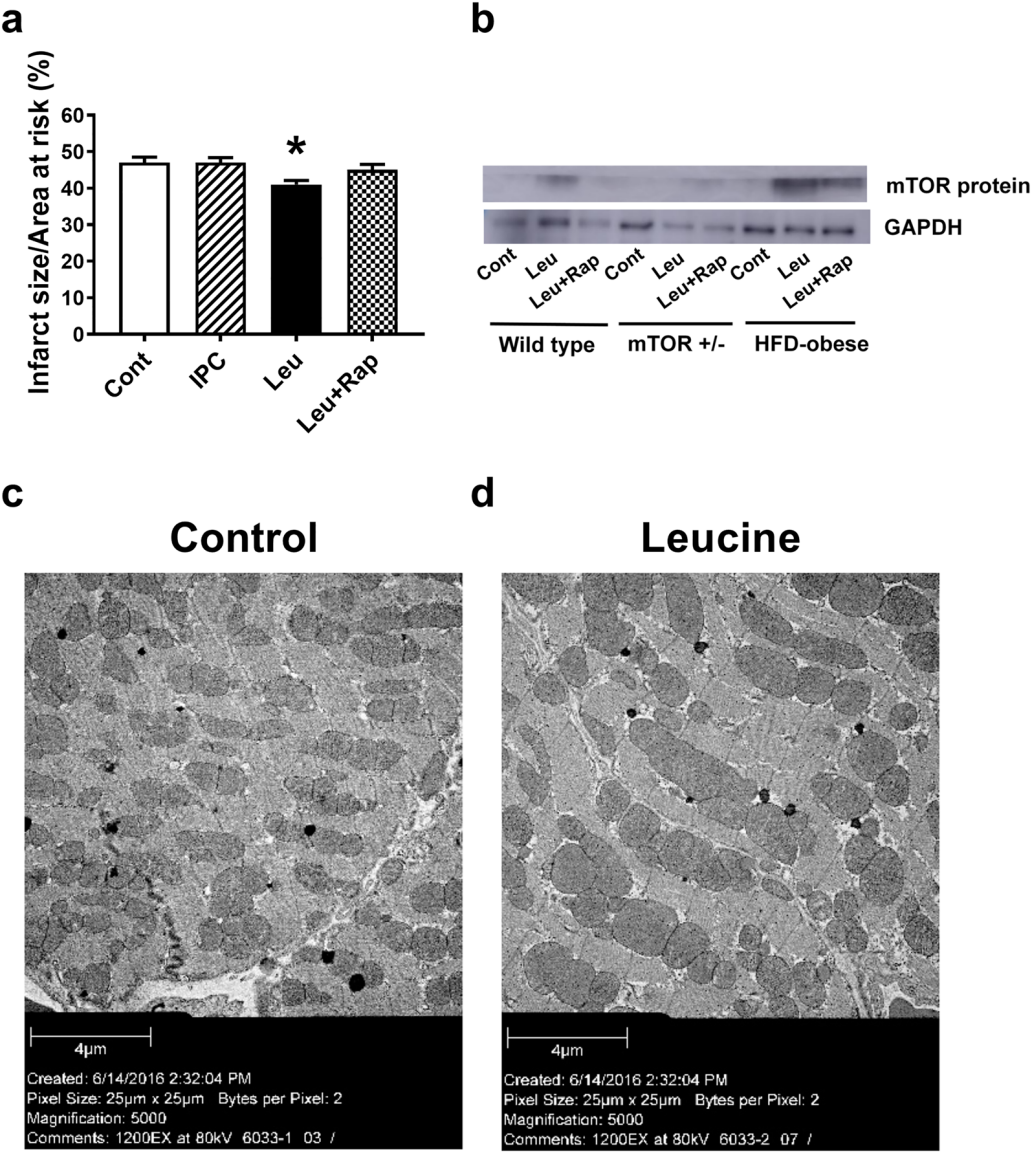
Effects of leucine (Leu) on ischemia/reperfusion (I/R) injury and mitochondrial fusion in high-fat diet (HFD)-induced obese mice. **a** HFD-induced obese mice were subjected to I/R. No significant difference in infarct size between the control (Cont) group and the ischemic preconditioning (IPC) group was observed, whereas Leu treatment reduced the infarct size caused by I/R injury in HFD-induced obese mice. The protective effect of Leu was abrogated by rapamycin (Rap). *p < 0.05, vs. Cont. **b** mTOR protein expression was examined in wild-type, mTOR^+/−^, and HFD-induced obese mice. Leu treatment increased mTOR expression in wild-type and HFD-induced obese mice, but not in mTOR^+/−^ mice. Leu, leucine; HFD, high-fat diet; mTOR, mammalian target of rapamycin; Rap, rapamycin; Cont, control; GAPDH, glyceraldehyde 3-phosphate dehydrogenase. **c** The changes in mitochondrial dynamics caused by Leu were determined using electron microscopy. Enhanced mitochondrial fusion was observed in the heart tissues of HFD-induced obese mice

In HFD-induced obese mice, IPC did not affect the infarct size caused by I/R injury (47.0 ± 1.4% vs. 47.0 ± 1.5%, n = 7, p > 0.999) (Fig. 3a). However, Leu treatment reduced the infarct size (41.0 ± 1.1% vs. 47.0 ± 1.5%, n = 7, p < 0.05), whereas this effect was not observed in the group administered with Leu and Rap (Fig. 3a).

### Leu enhanced mTOR protein expression

mTOR expression was enhanced by Leu administration in wild-type and HFD-induced obese mice, but not in mTOR^+/−^ mice (Fig. 3b). However, Rap inhibited the increase in mTOR expression in wild-type and HFD-induced obese mice.

### Mitochondrial dynamics under in vivo conditions

Mitochondrial dynamics were examined upon Leu administration in the heart tissue of HFD-induced obese mice (Fig. 3c) and we found that Leu administration enhanced mitochondrial fusion in the myocardium of HFD-induced obese mice.

### Leu decreased ROS production in wild-type and HFD-induced obese mice but not in mTOR^+/−^ mice

**Fig. 4:**
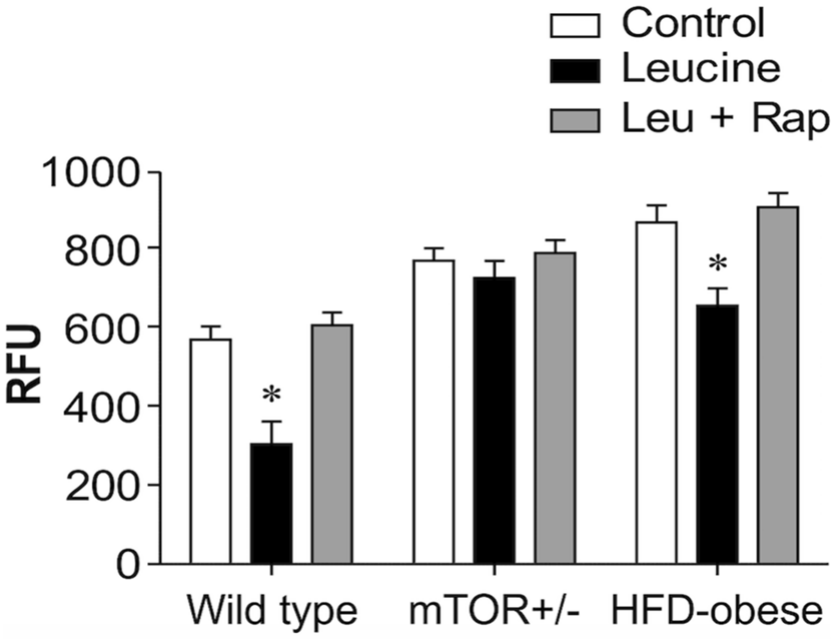
Reactive oxygen species (ROS) production in myocardium pretreated with leucine (Leu). ROS generation was examined after inducing ischemia/reperfusion injury in wild-type, mTOR^+/−^, and HFD-induced obese mice. Leu treatment resulted in decreased ROS generation in wild-type and HFD-induced obese mice, and this effect was inhibited by Rap. *p < 0.05, vs. control (Cont). mTOR, mammalian target of rapamycin; Rap, rapamycin; RFU, relative fluorescence units

After the I/R procedure, ROS production was decreased by Leu treatment in wild-type and HFD-induced obese mice but not in mTOR^**+/−**^ mice. Moreover, Rap inhibited the effect of Leu in wild-type and HFD-induced obese mice (Fig. 4).

## Discussion

Through several experimental approaches, we have provided new evidence suggesting that Leu reduces the infarct size caused by myocardial I/R injury in wild-type and HFD-induced obese mouse models. Additionally, Leu enhanced mitochondrial fusion after simulated I/R in rat cardiomyocytes. In mTOR^+/−^ mice, Leu did not affect infarct size, thereby suggesting that the effect of Leu was mediated through the mTOR signaling pathway. As observed in wild-type mice, Leu could impart protective effects on an HFD-induced obese mouse model by preconditioning treatment, which is not provided by IPC. Furthermore, Leu treatment led to an increase in mitochondrial fusion and a decrease in ROS production in prediabetic hearts.

Leu is a BCAA that plays a significant role in protein synthesis [26]. Previously, we showed that BCAAs have cardioprotective effects via the mTOR signaling pathway [12]. In present study, we found that Leu reduced infarct size in wild-type mice, but not in mTOR^+/−^ mice. Therefore, this result indicates that Leu is the key amino acid among BCAAs protects the heart from I/R injury through mTOR activity.

In this study, our finding indicates that Leu regulates the mTOR signaling pathway and improve mitochondrial dynamics, which means the increase in mitochondrial fusion. Mitochondrial dynamics and coordinated fission and fusion cycles are important for maintaining the shape, distribution, and size of mitochondria [27]. The Disintegration of the reticular form of mitochondria into fragments has been considered as a physiological indicator of mitochondrial dysfunction [28]. Several studies have shown that mTOR activity contributes to mitochondrial function [29]. Szabo et al. showed that mitochondrial fusion with mTOR phosphorylation had a preventive effect on mitochondrial fragmentation [30]. Further studies are, however, needed to identify the detailed signaling pathways involved between mTOR activity and mitochondrial dynamics in cardiac preconditioning induced by Leu treatment.

Mitochondria have been implicated as a major source of I/R-induced ROS production in a variety of organs [31]. Damage to mitochondria can change mitochondrial structure and function. These ultrastructural and functional defects partially recover upon reperfusion, and are accompanied by increased superoxide anion generation, resulting in a burst of ROS production upon reperfusion [32]. In our study, the decrease in ROS production was observed with Leu treatment, suggesting that Leu can prevent enhanced ROS production followed by the loss of mitochondrial function due to I/R injury in wild-type and HFD-induced obese mice.

In HFD-induced obese mice, we demonstrated that Leu reduces the infarct size, which is not observed by IPC. Cardiac preconditioning is attenuated by diabetes [33], and the loss of cardioprotective effects may be caused by the inhibition of insulin signaling whereas the effects of Leu seem not to largely depend on the insulin-related mechanisms [34]. Our previously study revealed that the effects of BCAA preconditioning are mediated via the mTOR pathway and not the phosphoinositide 3-kinase pathway, which is downstream of insulin receptor signaling [12]. Our results suggest that Leu is a key protein that recovers the effect of preconditioning, which was attenuated in case of IPC, through mTOR activity in prediabetic hearts.

This study has several limitations. First, we did not conduct experiments to investigate the molecular mechanism of enhanced mitochondrial fusion caused by Lue treatment for myocardial I/R injury. There are some proteins that relate to the process of mitochondrial fusion [35]. Identifying the specific protein could lead to the detail of pharmacological preconditioning mechanism by Leu via mTOR pathway in the future study. Second, we measured total ROS production in mice hearts. ROS is mainly produced by mitochondria but there are other ways to generate through several enzymatic reactions [31]. It might be more appropriate to measure mitochondrial ROS production to investigate the interaction between mitochondrial dynamics and ROS production.

## Conclusion

Leu treatment resulted in cardiac preconditioning with increased mTOR activity and mitochondrial fusion in both wild-type and HFD-induced obese mice, thereby suggesting that Leu could be potentially used for myocardial I/R injury treatment in patients with diabetes.

## Data Availability

The datasets used and analyzed during the current study are available from the corresponding author on reasonable request.

## Abbreviations

CAD: Coronary artery disease
AMI: Acute myocardial infarction
I/R: Ischemia/reperfusion
BCAA: Branched-chain amino acid
mTOR: Mammalian target of rapamycin
Leu: Leucine
HFD: High-fat diet
LCA: Left coronary artery
IPC: Ischemic preconditioning
ROS: Reactive Oxygen Species
Rap: Rapamycin
PBS: Phosphate-buffered saline
EM: Electron microscope

## References

[1] Beckman JA, Creager MA, Libby P (2002). Diabetes and atherosclerosis: epidemiology, pathophysiology, and management. JAMA.

[2] Ding M, Lei J, Han H, Li W, Qu Y, Fu E, Fu F, Wang X (2015). SIRT1 protects against myocardial ischemia-reperfusion injury via activating eNOS in diabetic rats. Cardiovasc Diabetol.

[3] Tanaka K, Kehl F, Gu W, Krolikowski JG, Pagel PS, Warltier DC, Kersten JR (2002). Isoflurane-induced preconditioning is attenuated by diabetes. Am J Physiol Heart Circ Physiol.

[4] Aguilar D, Solomon SD, Køber L, Rouleau JL, Skali H, McMurray JJ, Francis GS, Henis M, O'Connor CM, Diaz R (2004). Newly diagnosed and previously known diabetes mellitus and 1-year outcomes of acute myocardial infarction: the VALsartan In Acute myocardial iNfarcTion (VALIANT) trial. Circulation.

[5] Stevens RJ, Coleman RL, Adler AI, Stratton IM, Matthews DR, Holman RR (2004). Risk factors for myocardial infarction case fatality and stroke case fatality in type 2 diabetes: UKPDS 66. Diabetes Care.

[6] Li H, Liu Z, Wang J, Wong GT, Cheung CW, Zhang L, Chen C, Xia Z, Irwin MG (2013). Susceptibility to myocardial ischemia reperfusion injury at early stage of type 1 diabetes in rats. Cardiovasc Diabetol.

[7] Patel HH, Tsutsumi YM, Head BP, Niesman IR, Jennings M, Horikawa Y, Huang D, Moreno AL, Patel PM, Insel PA (2007). Mechanisms of cardiac protection from ischemia/reperfusion injury: a role for caveolae and caveolin-1. Faseb j.

[8] Tsutsumi YM, Yokoyama T, Horikawa Y, Roth DM, Patel HH (2007). Reactive oxygen species trigger ischemic and pharmacological postconditioning: in vivo and in vitro characterization. Life Sci.

[9] Ibáñez B, Heusch G, Ovize M, Van de Werf F (2015). Evolving therapies for myocardial ischemia/reperfusion injury. J Am Coll Cardiol.

[10] Tsutsumi YM, Kawaraguchi Y, Niesman IR, Patel HH, Roth DM (2010). Opioid-induced preconditioning is dependent on caveolin-3 expression. Anesth Analg.

[11] Stary CM, Tsutsumi YM, Patel PM, Head BP, Patel HH, Roth DM (2012). Caveolins: targeting pro-survival signaling in the heart and brain. Front Physiol.

[12] Satomi S, Morio A, Miyoshi H, Nakamura R, Tsutsumi R, Sakaue H, Yasuda T, Saeki N, Tsutsumi YM (2020). Branched-chain amino acids-induced cardiac protection against ischemia/reperfusion injury. Life Sci.

[13] Sanchez Canedo C, Demeulder B, Ginion A, Bayascas JR, Balligand JL, Alessi DR, Vanoverschelde JL, Beauloye C, Hue L, Bertrand L (2010). Activation of the cardiac mTOR/p70(S6K) pathway by leucine requires PDK1 and correlates with PRAS40 phosphorylation. Am J Physiol Endocrinol Metab.

[14] Kilkenny C, Browne WJ, Cuthill IC, Emerson M, Altman DG (2010). Improving bioscience research reporting: the ARRIVE guidelines for reporting animal research. PLoS Biol.

[15] Murakami M, Ichisaka T, Maeda M, Oshiro N, Hara K, Edenhofer F, Kiyama H, Yonezawa K, Yamanaka S (2004). mTOR is essential for growth and proliferation in early mouse embryos and embryonic stem cells. Mol Cell Biol.

[16] Tsutsumi YM, Horikawa YT, Jennings MM, Kidd MW, Niesman IR, Yokoyama U, Head BP, Hagiwara Y, Ishikawa Y, Miyanohara A (2008). Cardiac-specific overexpression of caveolin-3 induces endogenous cardiac protection by mimicking ischemic preconditioning. Circulation.

[17] Horikawa YT, Patel HH, Tsutsumi YM, Jennings MM, Kidd MW, Hagiwara Y, Ishikawa Y, Insel PA, Roth DM (2008). Caveolin-3 expression and caveolae are required for isoflurane-induced cardiac protection from hypoxia and ischemia/reperfusion injury. J Mol Cell Cardiol.

[18] Hirose K, Tsutsumi YM, Tsutsumi R, Shono M, Katayama E, Kinoshita M, Tanaka K, Oshita S (2011). Role of the O-linked β-N-acetylglucosamine in the cardioprotection induced by isoflurane. Anesthesiology.

[19] Tsutsumi YM, Kawaraguchi Y, Horikawa YT, Niesman IR, Kidd MW, Chin-Lee B, Head BP, Patel PM, Roth DM, Patel HH (2010). Role of caveolin-3 and glucose transporter-4 in isoflurane-induced delayed cardiac protection. Anesthesiology.

[20] Horikawa YT, Panneerselvam M, Kawaraguchi Y, Tsutsumi YM, Ali SS, Balijepalli RC, Murray F, Head BP, Niesman IR, Rieg T (2011). Cardiac-specific overexpression of caveolin-3 attenuates cardiac hypertrophy and increases natriuretic peptide expression and signaling. J Am Coll Cardiol.

[21] Matsushima R, Takahashi A, Nakaya Y, Maezawa H, Miki M, Nakamura Y, Ohgushi F, Yasuoka S (2006). Human airway trypsin-like protease stimulates human bronchial fibroblast proliferation in a protease-activated receptor-2-dependent pathway. Am J Physiol Lung Cell Mol Physiol.

[22] Parra V, Eisner V, Chiong M, Criollo A, Moraga F, Garcia A, Härtel S, Jaimovich E, Zorzano A, Hidalgo C (2008). Changes in mitochondrial dynamics during ceramide-induced cardiomyocyte early apoptosis. Cardiovasc Res.

[23] Yu T, Robotham JL, Yoon Y (2006). Increased production of reactive oxygen species in hyperglycemic conditions requires dynamic change of mitochondrial morphology. Proc Natl Acad Sci U S A.

[24] Tsutsumi YM, Tsutsumi R, Hamaguchi E, Sakai Y, Kasai A, Ishikawa Y, Yokoyama U, Tanaka K (2014). Exendin-4 ameliorates cardiac ischemia/reperfusion injury via caveolae and caveolins-3. Cardiovasc Diabetol.

[25] Tsutsumi YM, Patel HH, Lai NC, Takahashi T, Head BP, Roth DM (2006). Isoflurane produces sustained cardiac protection after ischemia-reperfusion injury in mice. Anesthesiology.

[26] Proud CG (2002). Regulation of mammalian translation factors by nutrients. Eur J Biochem.

[27] Tilokani L, Nagashima S, Paupe V, Prudent J (2018). Mitochondrial dynamics: overview of molecular mechanisms. Essays Biochem.

[28] Nagdas S, Kashatus DF (2017). The Interplay between Oncogenic Signaling Networks and Mitochondrial Dynamics. Antioxidants.

[29] de López KG, Toledo Guzmán ME, Sánchez EO, García CA (2019). mTORC1 as a Regulator of mitochondrial functions and a therapeutic target in cancer. Front Oncol.

[30] Szabo A, Sumegi K, Fekete K, Hocsak E, Debreceni B, Setalo G, Kovacs K, Deres L, Kengyel A, Kovacs D (2018). Activation of mitochondrial fusion provides a new treatment for mitochondria-related diseases. Biochem Pharmacol.

[31] Granger DN, Kvietys PR (2015). Reperfusion injury and reactive oxygen species: The evolution of a concept. Redox Biol.

[32] Lesnefsky EJ, Moghaddas S, Tandler B, Kerner J, Hoppel CL (2001). Mitochondrial Dysfunction in Cardiac Disease: Ischemia-Reperfusion, Aging, and Heart Failure. J Mol Cell Cardiol.

[33] Engbersen R, Riksen NP, Mol MJ, Bravenboer B, Boerman OC, Meijer P, Oyen WJ, Tack C, Rongen GA, Smits P (2012). Improved resistance to ischemia and reperfusion, but impaired protection by ischemic preconditioning in patients with type 1 diabetes mellitus: a pilot study. Cardiovasc Diabetol.

[34] Stipanuk MH (2007). Leucine and protein synthesis: mTOR and beyond. Nutr Rev.

[35] Westermann B (2010). Mitochondrial fusion and fission in cell life and death. Nat Rev Mol Cell Biol.

